# Retrospective analysis of the prognostic factors of fetal corpus callosum dysplasia

**DOI:** 10.1186/s12884-024-06300-w

**Published:** 2024-02-01

**Authors:** Ruina Huang, Junya Chen, Xinlin Hou, Lili Liu, Guoyu Sun, Hong Pan, Yinan Ma

**Affiliations:** 1 Department of Obstetrics and Gynecology, Peking University First Hospital, Beijing, 100034 China; 2 Department of Pediatrics, Peking University First Hospital, Beijing, 100034 China; 3 Department of Central Laboratory, Peking University First Hospital, Beijing, 100034 China

**Keywords:** Fetus, Dysplasia of the corpus callosum, Partial absence of the corpus callosum, Prenatal ultrasound

## Abstract

**Background:**

To analyze the genetic characteristics and long-term outcomes of fetuses with dysplasia of the corpus callosum (DCC) or partial agenesis of the corpus callosum (PACC).

**Methods:**

A total of 42 fetuses with DCC (*n* = 36) or PACC (*n* = 6) were retrospectively analyzed from January 2016 to December 2022 at the Peking University First Hospital. The cohort was categorized into isolated (15/42, 36%) and nonisolated groups (27/42, 64%), and differences in the genetic abnormalities and long-term outcomes between the two groups were analyzed. DCC was subdivided into short CC, thin CC, and thick CC. The outcomes of the three different types of DCC were analyzed and discussed.

**Results:**

(1) Thirty-nine of the 42 cases underwent CMA (chromosomal microarray analysis) and CMA + WES (whole exome sequencing), with 13/15 cases in isolated group and 26/27 cases in nonisolated group. Only pathogenic or likely pathogenic (P/LP) variants were considered, identifying P/LP variants in 2/13 cases in isolated group and 12/26 cases in nonisolated group. There was no significant difference between the two groups (χ² = 3.566, *P* = 0.05897). (2) In the isolated group, 8 cases were terminated, and 7 cases were delivered. Postnatal follow-up detected 1 case of gross motor development delay one year after birth; no obvious abnormalities were found in the other six cases. In the nonisolated group, 21 cases were terminated, and 6 cases were delivered. Postnatal follow-up detected 4 cases of children with different degrees of language, motor and intelligence abnormalities; 1 case died 10 days after birth. No obvious abnormalities were observed in one case. Six cases (86%, 6/7) in the isolated group showed normal development, compared with 1 case (17%, 1/6) in the nonisolated group, with a significant difference (χ² = 6.198, *P* = 0.01279). (3) In DCC, the delivery rates of short CCs (18 cases), thin CCs (13 cases), and thick CCs (5 cases) were 17% (3/18), 54% (7/13), and 20% (1/5), respectively, with good outcomes observed in 0% (0/3), 71% (5/7), and 0% (0/1), respectively. P/LP variants were found in 6/17 cases of short CC, 3/12 cases of thin CC, and 2/5 cases of thick CC.

**Conclusions:**

Fetuses with DCC or PACC combined with other structural abnormalities had a poor long-term prognosis compared with the isolated group. Patients with thin CCs had a higher probability of a good prognosis than those with short or thick CCs.

## Background

The incidence of abnormalities of the corpus callosum (CC) is approximately 1.8 per 100,000 in the general population and 230–600 per 100,000 in individuals with neurodevelopmental disorders [1, 2]. The long-term outcomes of fetuses with abnormalities of the CC are mainly related to the etiology of the condition and whether abnormalities of the CC appear isolated on imaging.

The prenatal diagnosis of agenesis of the CC (ACC) has been comprehensively described [3, 4]. Approximately two-thirds of children with isolated complete agenesis of the CC (CACC) have good developmental outcomes, but these children may have different degrees of defects that become more obvious between 10 and 20 years of age and include neuropsychological disorders and behavioral disorders [5]. Children with partial agenesis of the CC (PACC) or nonisolated ACC have worse later-life prognoses [4, 6]. However, there is limited information on short, thin, or thick CCs [7–9]. The wide range of neurodevelopmental presentations associated with ACC indicates the necessity of the accurate assessment and diagnosis of fetuses to better inform prenatal counseling.

In this study, we classified short, thin, and thick CCs as DCCs and retrospectively analyzed the clinical data, pregnancy outcomes and neurodevelopmental outcomes of children with antenatal diagnoses of PACC and DCC in our center. Factors influencing fetal CC abnormalities were also analysed. These data provide evidence for the clinical consultation and management of fetal PACC and DCC.

## Methods

### Research subjects

**Fig. 1:**
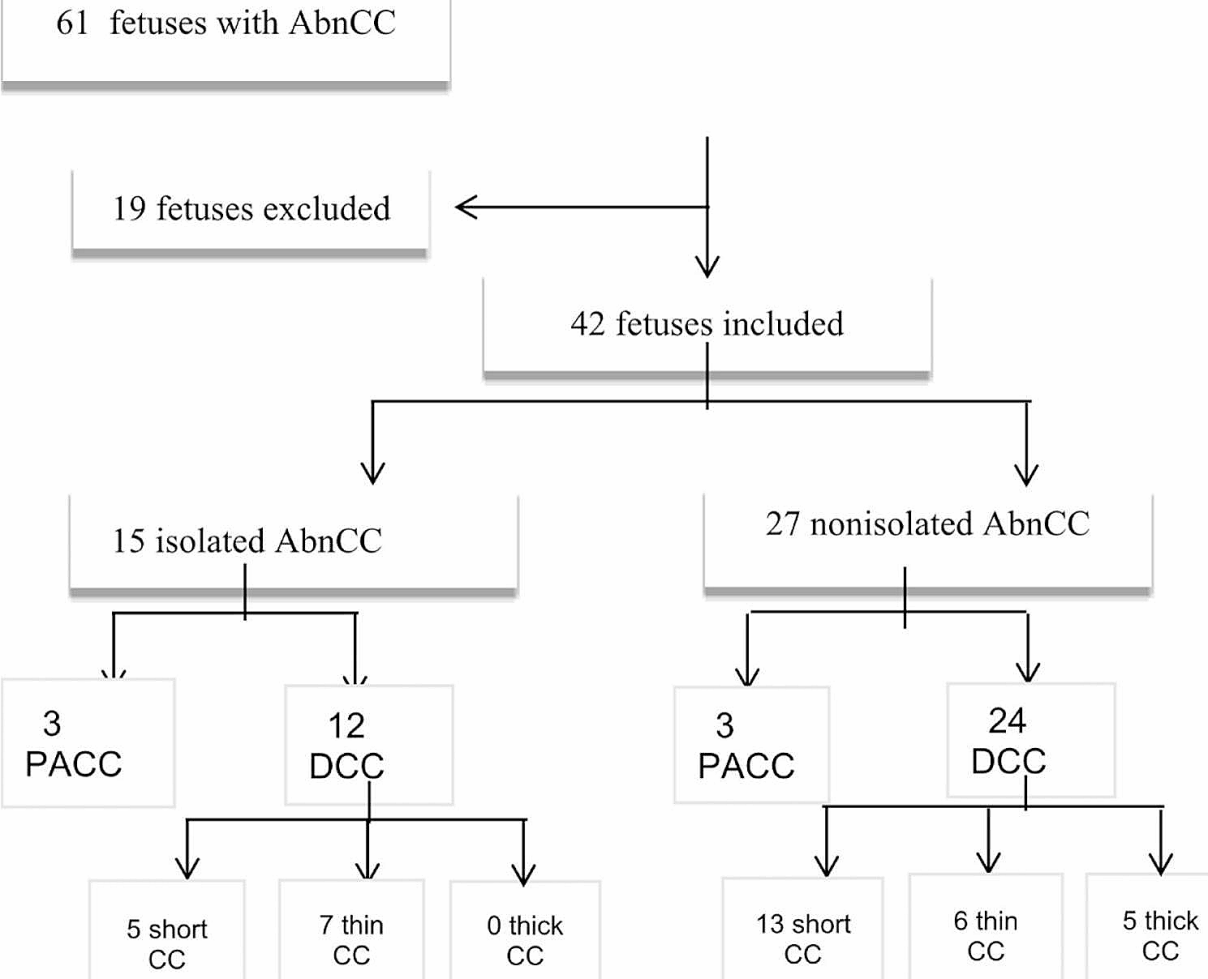
Flowchart of the study

Abbreviations: AbnCC: abnormalities of the corpus callosum; PACC: partial agenesis of the corpus callosum; DCC: dysplasia of the corpus callosum; CC: corpus callosum.

From January 2016 to December 2022, 61 fetuses were diagnosed with fetal PACC or DCC by ultrasound at the Peking University First Hospital. The inclusion criterion for the study was a gestational age ≥ 20 weeks at diagnosis with clear neurosonographic (NSG) images. Fetuses with no imaging data or poor-quality imaging data were excluded to allow a detailed analysis of the brain, including the CC. Finally, 42 fetuses were included in the study, of which 36 fetuses were diagnosed with DCC and 6 fetuses were diagnosed with PACC. The study flowchart is shown in Fig. 1. Since this study was a retrospective study, no patient information was exposed in the process of case collection, data analysis and paper writing. This study was granted exemption by the ethics committee of Peking University First Hospital (approval no.2022yan249−002).

### Instruments and methods

Ultrasound examinations were conducted using a Voluson E8 or E10 ultrasound device (GE Healthcare Ultrasound, Milwaukee, WI). Transabdominal and transvaginal NSGs were performed by experienced doctors using 3–5 MHz, 1–7 MHz, 5–9 MHz, and 6–12 MHz probes to define central nervous system (CNS) abnormalities. The NSG examination was performed according to the International Society of Ultrasound in Obstetrics and Gynecology (ISUOG) guidelines [10]. If there was a head presentation and no other risk factors, such as bleeding, premature rupture of membranes, or threatened preterm birth, transvaginal NSG was preferred. If the presentation was breech, only transabdominal NSG could be performed. Comprehensive scans for extracranial abnormalities were routinely performed.

The PACC diagnostic criteria using NSG included incomplete or partially absent CC in the midsagittal and coronal planes. In the case of PACC, the missing part (rostrum, genu, body, or splenium) of the CC was recorded.

Ultrasonographic findings of DCC: A short CC was defined as a complete CC with an anteroposterior diameter below the third percentile compared with a complete CC [7, 8, 11]. Thick and thin CCs were assessed visually by experienced experts, also comparing about ten different fetuses at the same GA not referred for brain malformations, used as control cases [7, 12].

The 42 enrolled cases were classified into nonisolated and isolated groups according to the presence or absence of other cerebral or extracerebral malformations [7, 13–15]. Based on the morphology of the CC, DCC was classified into short, thin, and thick CCs.

With the permission of the mother, genetic examinations were performed on the fetuses. All samples of amniotic fluid were subjected to karyotype and chromosomal microarray (CMA). Samples negative for CMA were subjected to whole-exome sequencing (WES) with the parents’ permission. Data were analyzed using CytoGenomics (version 5.0.2.5) software. The Trio-WES strategy was used to identify variants from family trees. Library preparation was performed using Illumina Library Amplification, and HiFi HotStart ReadyMix (KAPA) was used for library amplification. The NovaSeq 6000 platform (Illumina, USA) with a 150 bp paired-end sequencing mode was used to sequence the genomic DNA of the family. Raw data were analyzed using NextGENe software (version 2.4.2.3, SoftGenetics, USA). The GRCh38 genome was used for annotation. All identified variants were further analyzed with reference to public databases, including ClinGen, DGV, gnomAD, the 1000 Genome Project, DECIPHER, ClinVar, OMIM, and a comprehensive review of literature from PubMed to determine their clinical significance. The variants were classified into five categories, namely, pathogenic, likely pathogenic, uncertain significance, likely benign, and benign, according to the American College of Medical Genetics and Genomics (ACMG) guidelines for the interpretation of genetic variants [16, 17]. Only pathogenic (P) and likely pathogenic (LP) variants were included in the study.

Postnatal follow-up was performed using the Ages and Stages Questionnaire, Third Edition (ASQ-3). Child development was assessed by verbal communication along with gross and fine motor skills.

### Statistical analysis

Descriptive analysis was performed on all data. Measurement data are expressed as the mean ± standard deviation, and count data are expressed as the frequency and rate. Data were compared between the groups using a Chi squared test. *P* values of < 0.05 were considered statistically significant.

## Results

### General Information

Among the 42 cases, 36 were DCC and 6 were PACC. The mean gestational age and mean age of the 42 pregnant women were 25.7 ± 3.1 weeks (21–37 weeks) and 30.2 ± 4.5 years (21–39 years), respectively. Of the 42 cases, 29 (69%) cases terminated pregnancies, and 13 cases (31%) continued pregnancy until delivery.

### Associated malformations of fetuses with CC abnormalities

The isolated group included 15 fetuses, with 27 fetuses in the nonisolated group, including 20 cases with intracranial structural malformations, 2 cases with intracranial cysts, 1 case with pericallosal lipoma, 1 case with Dandy-Walker malformation, 1 case with lobar holoprosencephaly, 7 cases with malformations of cortical development (MCD), 1 case with schizencephaly, 2 cases with cerebral hemorrhage, 4 cases with cerebral ventriculomegaly, and 1 case with microcephaly. Seven cases were complicated with extracranial malformations, including 1 case with a high-arched palate, 1 case with micrognathia, 2 cases with congenital heart defects, 1 case with a narrow thoracic vertebral canal, 1 case with limb abnormalities, and 1 case with multiple malformations.

### Magnetic resonance imaging (MRI) results

Thirty-six cases underwent prenatal MRI examinations, and 34 cases were consistent with an MRI diagnosis with a coincidence rate of 94% (34/36); the other two cases showed additional evidence of MCD on MRI.

### Fetal genetic results and pregnancy outcome analysis

Apart from 3 cases where genetic examination was refused, 39 of the 42 cases underwent genetic examinations. Eight cases underwent CMA only, and 5 cases underwent CMA + WES in the isolated group, while 13 cases underwent CMA only, and 13 cases underwent CMA + WES in the nonisolated group.

In the isolated group, 13 of the 15 cases underwent genetic examination, while LP variants were detected by WES in 2 cases. In 1 case, a suspected pathogenic variant was detected in the *ARIDIB* gene that was associated with autistic spectrum disorder. In another case, a likely pathogenic variant was detected in the *PTEN* gene. Genetic examination was refused in 2 cases. Pregnancies were terminated in 8 cases, while 7 cases were delivered.

In the nonisolated group, 26 of 27 cases underwent genetic examinations, and 12 cases were found to have P or LP variants, including 1 case of trisomy 18 and 7 cases detected by WES. Genetic examination was refused in 1 case. Pregnancies were terminated in 21 cases, while 6 cases were delivered. The fetal genetic results and pregnancy outcomes of the two groups are summarized in Table 1.

**Table 1:** Summary of the fetal genetic results and pregnancy outcomes in the two groups [cases (%)]

group	number of cases	PACC	DCC			cases with genetic tests	cases with P or LP genetic results/total (%)	termination/total (%)	delivery		
			short	thin	thick				good outcome/total (%)	poor outcome/total (%)	
Isolation group	15	3	5	7	0	13	2 2/13 (15%)	8	6(86%)	1(14%)	
Nonisolated group	27	3	13	6	5	26	12 12/26 (46%)	21	1(17%)	5(83%)	
X2 value *P* values							3.566 0.05897		6.198 0.01279		

PACC: partial agenesis of the corpus callosum; DCC: dysplasia of the corpus callosum; P: pathogenic; LP: likely pathogenic

### Follow-up information

Of the 15 fetuses in the isolated group, 7 were delivered. The follow-up duration ranged from 4 to 18 months after birth. One child suffered from delayed motor development and did not receive rehabilitation treatment. No obvious delays in growth and development or intellectual disabilities were found in the other 6 cases. Among the 27 fetuses in the nonisolated group, 6 were delivered. The follow-up duration ranged from 8 to 24 months. Case 6 had continuous excessive dorsiflexion of both ankle joints, case 7 experienced epileptic seizures, two cases (cases 8 and 10) had ambiguous articulation and delayed motor development, and case 9 suffered from dysphagia and died 10 days after birth. No obvious developmental abnormalities were observed in case 11. The postnatal development of the fetuses is summarized in Table 2.

**Table 2:** Summary of the postnatal follow-up of 10 fetuses with an abnormal corpus callosum (CC)

case	diagnostic GA	Diagnosis of CC	other abnormalities	CMA	WES	Follow-up duration(months after birth)	Postnatal outcomes
1	33	thin CC	NO	NO	NO	16	normal
2	21	thin CC	NO	normal	NO	8	gross movements were more sluggish
3	23	thin CC	NO	normal	NO	18	normal
4	37	the rostral absent	NO	NO	NO	3	normal
5	25	the splenium absent	NO	normal	NO	5	normal
6	36	short CC	Cerebral hemorrhage	NO	normal	12	continuous excessive dorsiflexion of both ankle joints
7	34	thin CC	lateral ventriculomegalyand the ventricular wall was irregular	normal	NO	12	epilepsy and delayed motor development
8	25	thick CC	MCD	normal	NO	9	ambiguous articulation and delayed motor development
9	38	short CC	microcephaly	A possible pathogenic CNV of 29.76 Mb was identified at del(5)(p15.33p13.3)(chr5:24 261-2978284 6) in seq [GRCH37]			died 10 days after birth
10	32	short CC	Severe ventriculomeg-aly	normal	A variant in the ADNP gene was identified and associated with the following diseases: HELSMOORTEL-VAN DER AA syndrome	24	ambiguous articulation and delayed motor development, poor rehabilitation treatment
11	37	thin CC	pericallosal lipoma	normal	normal	8	normal
12	34	thin CC	NO	normal	normal	4	normal
13	26	thin CC	NO	normal	normal	8	normal

CC: corpus callosum; CMA: chromosomal microarray analysis; WES: whole exome sequencing;

GA; gestational age (weeks)

### Comparison of the 36 fetuses with DCC

Based on the morphology of the fetal CC, 36 DCC fetuses were classified into three groups, namely, 18 cases (50%) with short CCs, 13 cases (36%) with thin CCs, and 5 cases (14%) with thick CCs. The combined malformations, genetic abnormalities, and pregnancy outcomes associated with the different morphological combinations were compared and are summarized in Table 3.

Four cases were born with a short or thick CC, all of which were associated with other malformations and had a poor prognosis. Seven cases were born with a thin CC, of which 5 cases were isolated and 1 case had a poor prognosis, while the remaining 2 cases were nonisolated and 1 case had a poor prognosis.

**Table 3:** Comparison of factors associated with different morphologies of the corpus callosum

different morphology of CC	number	nonisolated (%)	genetic testing	PorLP genetic results(%)	termination	born		
						good outcomes	poor outcomes	
*short*	18	13(72%)	17	6 (6/17,35%)	15	0	3(100%)	
thick	5	5(100%)	5	2(2/5,40%)	4	0	1(100%)	
thin	13	6(46%)	12	3(3/12,25%)	6	5	2(29%)	

CC: corpus callosum; P: pathogenic; LP: likely pathogenic

## Discussion

The CC is located at the bottom of the longitudinal fissure of the cerebral hemisphere. It is the largest bundle of nerve fibers connecting the bilateral cerebral hemispheres and plays an important role in nerve conduction for human growth and development [18]. The development of the nervous system begins 23 days after fertilization, and the CC gradually develops approximately 51 days after fertilization [19]. The basic structure of the CC is completed at 18–20 weeks of gestation and continues to grow throughout the third trimester [8].

The diagnosis of abnormal development of the CC can only be made after 20 weeks of gestation. The gestational age of all cases in this study was > 20 weeks, with an average of 25.7 ± 3.1 weeks. A diagnosis of CC dysplasia cannot be obtained using axial views of the fetal brain. Only the midsagittal view can show the whole picture of the CC to confirm a diagnosis of dysplasia. When abnormalities of the fetal nervous system were suspected on regular ultrasound, NSG was further performed in this study. NSG, especially transvaginal NSG, is the gold standard for the diagnosis of fetal corpus callosum dysplasia [10]. In addition, many other fetal intracranial malformations can also be diagnosed by NSG. Among the 27 cases of nonisolated PACC and DCC, 19 cases were found to be associated with intracranial malformations, including schizencephaly, hydrocephalus, intracranial cysts, Dandy-Walker malformations and MCD. MRI is better able to diagnose fetal MCD. In this study, the combined application of NSG and MRI evaluated by experts guarantees the accurate diagnosis of fetal intracranial malformations.

Although it is possible to assess the development of the corpus callosum before birth, prenatal counseling for CC abnormalities is difficult [20]. The main reason is that there is a lack of prospective large sample studies to accurately explain its prognosis. The influencing factors also need further study. This study focused on the prognostic factors of fetuses with PACC and DCC and analyzed the likelihood of combinations with other malformations and the morphology of CC dysplasia affecting the fetal prognosis.

Genetic factors are one of the most common causes of CC abnormalities [21]. The incidence of monogenic disorders is 35%, that of genetic syndromes is 45%, and that of chromosomal abnormalities is 18%, mainly trisomy 18, 13 and mosaicism 8 [1]. Therefore, genetic investigations play a pivotal role in the workup of abnormalities of the CC. In this study, 39 cases underwent genetic testing, of which pathogenic or likely pathogenic variants were found in 14 (14/39, 35.9%). Of these 14 variants, 1 case showed chromosomal karyotype abnormalities (trisomy 18), 4 cases had pathogenic copy number variations, and 9 cases were detected by WES. No significant differences in genetic abnormalities were seen between the isolated and nonisolated groups, although the *P* value was 0.05897, very close to 0.05. In future research, expansion of the sample size is likely to result in a significant difference.

Previous studies have reported that children with nonisolated CC abnormalities often present with mental retardation, delayed neurodevelopment, poor motor and expression abilities, refractory epilepsy, and hypotonia after birth [22, 23]. More than 70% of children with isolated CC abnormalities have good or lower-limit intelligence and development of other systems [24]. However, many uncertainties remain concerning the long-term prognosis of isolated CC abnormalities, and long-term follow-up is needed to clarify a final prognosis. Thirteen of the 42 fetuses with CC abnormalities were born, including 7 cases of isolated DCC and 6 cases of nonisolated DCC. Seven cases in the isolated group were assessed at follow-up between 3 and 18 months after birth. Of these 7 cases, only one had slight movement retardation, and the other 6 cases (6/7, 86%) showed normal development. Six cases in the nonisolated group were all DCC with an age of 9 to 24 months after birth. Among these 6 cases, 5 suffered from different types and degrees of abnormal motor and language development after birth or died after birth. Only one case showed no significant developmental abnormalities at 8 months of age. Therefore, the fetuses with nonisolated abnormalities of the CC had a higher probability of a poor long-term prognosis than those with isolated abnormalities of the CC.

In addition to genetic abnormalities, it has been suggested that the type of abnormality of the CC could also impact the prognosis [1, 25–27], but due to different and unclear definitions in the literature, this point requires clarification. At present, there is no consensus on the classification of abnormal development of the corpus callosum. The classification of corpus callosum abnormalities in the present study was based on these two references [7, 21]. We defined short CC as complete with an anteroposterior diameter below the third percentile, with short, thin, and thick CC classified as DCC [7]. Nevertheless, the distinction between PACC and DCC can be very challenging and requires a thorough analysis of the CC.

In this study, 36 cases were prenatally diagnosed with DCC. Twenty-three cases had short or thick CCs, with more than 70% associated with malformations, while 13 cases with thin CCs had a 46% association with malformations. DCC was present in 11 of the 13 delivered fetuses, among which the probability of a good prognosis was zero for those with a short or thick CC and 71% for those with a thin CC.

Poor prognoses have been reported for a thick CC with abnormal head circumference and/or related malformations, and the significance of an isolated thick CC is unclear [8, 9, 28]. Our data suggest that short or thick CCs are more strongly associated with fetal malformations, genetic abnormalities and a poor prognosis, which should be given adequate attention during prenatal diagnosis and consultation. The cases of thick CCs were all nonisolated in this study. Few studies have addressed the different morphologies of fetal CCs, which is precisely the significance of this study. In the isolated group, thin CCs were associated with a relatively optimistic prognosis. However, more data are needed to verify these findings, and whether isolated thin CCs can be used to diagnose CC dysplasia remains to be discussed.

This study had several limitations. Most fetuses in this study were terminated in this study, preventing an accurate evaluation of their outcomes. The length of follow-up was also highly variable. Therefore, for infants with only one postnatal evaluation, their outcomes may not accurately reflect developmental delays since the age may have been too young to observe the progression of developmental milestones, which may underestimate the effect of CC dysplasia on neurodevelopment. Expanding the sample size, together with the standardization of neurological and developmental assessments in a prospective longitudinal study, would help to better understand the full clinical spectrum of outcomes of children with CC abnormalities.

## Conclusions

The risk of genetic abnormalities and poor short-term prognosis in the fetuses with nonisolated CC dysplasia was significantly higher than that in the fetuses with isolated CC dysplasia. Limited data show that in DCC cases, the risk of genetic abnormalities and poor short-term prognosis in fetuses with short or thick CCs is significantly higher than that in fetuses with a thin CC. Thus, for fetuses with CC abnormalities, the key to prenatal examination and consultation is to identify other intracranial and extracranial malformations by NSG and MRI and pay specific attention to the morphology of the CC. CMA combined with WES is preferred for prenatal genetic examination.

## Data Availability

The data that support the findings of this study are available on request from the corresponding author. The data are not publicly available due to privacy or ethical restrictions.

## Abbreviations

AbnCC: Abnormalities of the corpus callosum
ACC: Genesis of the corpus callosum
CACC: Complete agenesis of the corpus callosum
CC: Corpus callosum
CMA: Chromosomal microarray analysis
DCC: Dysplasia of the corpus callosum
GA: Gestational age (weeks)
LP: Likely pathogenic
P: Pathogenic
PACC: Partial agenesis of the corpus callosum
WES: Whole-exome sequencing

## References

[1] Edwards TJ, Sherr EH, Barkovich AJ, Richards LJ (2014). Clinical, genetic and imaging findings identify new causes for corpus callosum development syndromes. Brain.

[2] Glass HC, Shaw GM, Ma C, Sherr EH (2008). Agenesis of the corpus callosum in California 1983-2003: a population‐based study. Am J Med Genet.

[3] Paul LK (2011). Developmental malformation of the corpus callosum: a review of typical callosal development and examples of developmental disorders with callosal involvement. J Neurodev Disord.

[4] D’Antonio F, Pagani G, Familiari A (2016). Outcomes associated with isolated agenesis of the corpus callosum: a meta-analysis. Pediatrics.

[5] Romaniello R, Arrigoni F, De Salvo P (2021). Long-term follow-up in a cohort of children with isolated corpus callosum agenesis at fetal MRI. Ann Clin Transl Neurol.

[6] Sotiriadis A, Makrydimas G (2012). Neurodevelopment after prenatal diagnosis of isolated agenesis of the corpus callosum: an integrative review. Am J Obstet Gynecol.

[7] Nguyen T, Heide S, Guilbaud L, Valence S (2023). Abnormalities of the corpus callosum. Can prenatal imaging predict the genetic status? Correlations between imaging phenotype and genotype. Prenat Diagn.

[8] Bardin R, Leibovitz Z, Mashiach R (2022). Short and thick corpus callosum - the thin border between a minor anatomical variant to very poor outcome. J Matern Fetal Neonatal Med.

[9] Shinar S, Har-Toov J, Lerman-Sagie T (2016). Thick corpus callosum in the second trimester can be transient and is of uncertain significance. Ultrasound Obstet Gynecol.

[10] Paladini D, Malinger G, Birnbaum R (2021). ISUOG Practice guidelines (updated): sonographic examination of the fetal central nervous system. Part 2: performance of targeted neurosonography. Ultrasound Obstet Gynecol.

[11] Cignini P, Padula F, Giorlandino M (2014). Reference charts for fetal corpus callosum length: a prospective cross-sectional study of 2950 fetuses. J Ultrasound Med off J Am Inst Ultrasound Med.

[12] Izzo G, Toto V, Doneda C (2021). Fetal thick corpus callosum: new insights from neuroimaging and neuropathology in two cases and literature review. Neuroradiology.

[13] Raile V, Herz NA, Promnitz G (2020). Clinical outcome of children with Corpus Callosum Agenesis. Pediatr Neurol.

[14] Bernardes da Cunha S, Carneiro MC, Miguel Sa M (2021). Neurodevelopmental outcomes following prenatal diagnosis of isolated Corpus Callosum Agenesis: a systematic review. Fetal Diagn Ther.

[15] D’Ambrosio V, Boccherini C, Manganaro L (2021). Hypoplasia of the Corpus Callosum: a single Center experience and a Concise Literature Review. Fetal Pediatr Pathol.

[16] Richards S, Aziz N, Bale S, ACMG Laboratory Quality Assurance Committee (2015). Standards and guidelines for the interpretation of sequence variants: a joint consensus recommendation of the American College of Medical Genetics and Genomics and the Association for Molecular Pathology. Genet Med.

[17] Riggs ER, Andersen EF, Cherry AM (2020). Technical standards for the interpretation and reporting of constitutional copy-number variants: a joint consensus recommendation of the American College of Medical Genetics and Genomics (ACMG) and the Clinical Genome Resource (ClinGen). Genet Med.

[18] Aboitiz F, Montiel J (2003). One hundred million years of interhemispheric communication: the history of the corpus callosum. Braz J Med Biol Res.

[19] Muller F. The embryonic human brain: 3th edition [M]. WileyLisss, 2006: 12, 113, 294.

[20] ENSO Working Group (2021). Role of prenatal magnetic resonance imaging in fetuses with isolated anomalies of corpus callosum: multinational study. Ultrasound Obstet Gynecol.

[21] Leombroni M, Khalil A, Liberati M, D’Antonio F (2018). Fetal midline anomalies:diagnosis and counseling part 1:corpus callosum anomalies. Eur J Paediatr Neurol.

[22] Pilu G, Sandri F, Perolo A (1993). Et a1.Sonography of fetal agenesis of the corpus callosum: a survey of35 cases. Ultrasound Obstet Gynecol.

[23] Erocle C, Girard N, Cravello L (1998). Et a1.Prenatal diagnosis of fetal corpus callosm agenesis by ultrasonography and magnetic resonance imaging. Prenat Diagn.

[24] Ozyuncu O, Yazicioglu A, Turgal M (2014). Antenatal diagnosis and outcome of agenesis of corpus callosum: a retrospective review of 33 cases. Turk Ger Gynecol Assoc.

[25] Ghi T, Carletti A, Contro E (2010). Prenatal diagnosis and outcome of partial agenesis and hypoplasia of the corpus callosum. Ultrasound Obstet Gynecol.

[26] Lerman-Sagie T, Ben‐Sira L, Achiron R (2009). Thick fetal corpus callosum: an ominous sign?. Ultrasound Obstet Gynecol.

[27] Bartholmot C, Cabet S, Massoud M (2021). Prenatal imaging features and postnatal outcome of short corpus callosum: a series of 42 cases. Fetal Diagn Ther.

[28] Andronikou S, Pillay T, Gabuza L, Mahomed N, Naidoo J, Hlabangana LT, du Plessis V, Prabhu SP (2015). Corpus callosum thickness in children: an MR pattern-recognition approach on the midsagittal image. Pediatr Radiol.

